# Type-2 Inflammation in Health and Disease: Prevalence, Risk Factors and Multimorbidity

**DOI:** 10.3390/jcm13226662

**Published:** 2024-11-06

**Authors:** Charmaine J. M. Lim, Christoph Gross, Marie-Kathrin Breyer, Robab Breyer-Kohansal, Emiel F. M. Wouters, Sylvia Hartl

**Affiliations:** 1 Ludwig Boltzmann Institute for Lung Health, 1140 Vienna, Austria; charmaine.helk-lim@leadstudy.at (C.J.M.L.);; 2 Faculty of Medicine, Sigmund Freud Private University, 1020 Vienna, Austria; 3 Department of Respiratory and Pulmonary Diseases, Clinic Penzing, Vienna Healthcare Group, 1140 Vienna, Austria; 4 Department of Respiratory and Pulmonary Diseases, Clinic Hietzing, Vienna Healthcare Group, 1130 Vienna, Austria; 5 Nutrition and Translational Research in Metabolism (NUTRIM), Maastricht University Medical Center, 6229 ER Maastricht, The Netherlands

**Keywords:** fractional exhaled nitric oxide, blood eosinophil counts, pulmonary function, extrapulmonary, COPD introduction

## Abstract

**Background:** In patients with airflow obstruction, the levels of biomarkers of Type-2 (T2) inflammation serve to predict the effectiveness of inhaled corticosteroid and biological therapies. Elevated biomarkers of T2 inflammation, including fractional exhaled nitric oxide (FeNO, ≥20 ppb) and blood eosinophil counts (BEC, ≥300 cells/µL), were investigated in a population-based cohort of the Austrian LEAD study. **Methods:** A total of 4976 individuals (aged 18–82 years) were categorised into four groups based on their FeNO and BEC levels: normal with FeNO < 20 ppb and BEC < 300 cells/µL (n = 2634); FeNO ≥ 20 ppb only (n = 1623); BEC ≥ 300 cells/µL only (n = 340); and FeNO ≥ 20 ppb and BEC ≥ 300 cells/µL (n = 379). **Results:** In age- and sex-adjusted regression models, individuals with elevated BEC only were most associated with chronic cough and sputum production (odds ratios [95% CI]: 1.22 [0.78, 1.84] and 1.37 [1.13, 2.62], respectively), whilst individuals with both elevated T2 biomarkers were most associated with wheezing, dyspnoea and asthma (odds ratios [95% CI]: 2.27 [1.56, 3.26], 1.32 [0.64, 2.50] and 3.63 [2.69, 4.88] respectively). Elevated levels of both FeNO and BEC presented an additive effect in extrapulmonary conditions, particularly in allergy, eczema and rhino conjunctivitis (odds ratios [95% CI]: 2.30 [1.84, 2.88], 1.37 [1.03, 1.81] and 2.95 [2.34, 3.70], respectively). **Conclusions:** T2 inflammation marked by elevated levels of FeNO and/or BEC is not only associated with respiratory conditions but also extends to extrapulmonary characteristics, with an additive effect.

## 1. Introduction

Eosinophils are best known for their role in the body’s immune response and in combating parasitic, bacterial and viral infections. Recent insights reveal that these cells are additionally involved in several homeostatic processes, including metabolism, tissue remodelling and development, neuronal regulation, and epithelial and microbiome regulation. Eosinophils play pathologic roles in different diseases, particularly in asthma, chronic obstructive pulmonary disease (COPD) and chronic rhinosinusitis with nasal polyps. Novel biologic therapies targeting eosinophil maturation have since become available for specific therapeutic interventions in addition to the traditional nonspecific eosinophilic attenuation with glucocorticoids [1].

Eosinophils have additionally been considered to function as the effector arm of Type-2 (T2) immunity. More recent findings reveal the dynamic, self-perpetuating interplay between eosinophils and T2 lymphocytes in which eosinophils contribute to the initiation and effector functions of T2 immunity [2]. Jointly, blood eosinophil counts (BECs) and fractional exhaled nitric oxide (FeNO) have been suggested to be potential biomarkers to determine T2 inflammation despite being regulated via different inflammatory pathways [3,4,5,6,7]. Previous studies have investigated the role of both elevated FeNO and elevated BEC in chronic airway disease in the general population [8,9].

The current study aimed to assess the prevalence of T2 inflammation, characterised by elevated BEC and/or FeNO in the general population, and to identify clinical traits present in individuals with elevated levels of both biomarkers. Considering the pleotropic homeostatic roles of eosinophils and eosinophil-mediated processes across different tissues and organs, the potential involvement in multimorbidity was analysed.

## 2. Methods

### 2.1. Study Design and Study Population

The study included 4976 participants, aged ≥ 18 years, of the Austrian Lung, hEart, sociAl, boDy (LEAD) cohort study, conducted in Vienna and Lower Austria (clinicaltrials.gov; NCT017257518). All participants in this study had interpretable and valid FeNO, BEC and spirometric measurements at a second examination. All participants completed a comprehensive questionnaire, underwent physical examinations and provided blood for biochemical analysis. The design of the LEAD study and related methodology has been published elsewhere [10] and was approved by the local ethics committee of Vienna (EK-11-117-0711). All participants provided written, informed consent, and all methodologies were in accordance with the Declaration of Helsinki.

### 2.2. Blood Eosinophils and Fractional Exhaled Nitric Oxide

White blood cell counts were measured on fasted venous blood samples and reported with other leukocyte subpopulations. A BEC of <300 cells/µL was considered to be normal and ≥300 cells/µL to be elevated [4,11,12]. This is the consensus threshold provided by the Global Initiative for Asthma (GINA) guidelines and associated with significant disease severity and increased risk of exacerbations in various pulmonary diseases. FeNO was determined using a standardised FeNO device (NIOX VERO, Circassia Pharmaceuticals plc, Oxford, UK) in accordance with recommendations from the European Respiratory Society (ERS) and American Thoracic Society (ATS) [11,13]. FeNO levels of <20 ppb were considered normal and ≥20 ppb elevated [14].

### 2.3. Lung Function Measurements

Lung function in the present study included post-bronchodilator spirometry (forced expiratory volume in 1 second, FEV1; and forced vital capacity, FVC; BT-MasterScope Body 0.478, Jaegar, Germany; SentrySuite software version 3.20.1), and predicted lung function parameters were obtained with the Global Lung Initiative (GLI) reference equations [15]. The quality control of spirometry procedures was ensured by trained personnel and standard operating procedures. For an in-depth analysis with relevance to obstructive spirometric patterns, participants were further categorised based on their FEV1/FVC ratio to the lower limit of normal (LLN; z-scores, 1.645), for which a FEV1/FVC ratio < LLN is representative of airflow limitation, and positive reversibility is defined as the difference between post-bronchodilator and pre-bronchodilator FEV1 ≥ 200 mL and ≥12%. Individuals were also further stratified based on their smoking status for chronic airway disease (CAD; self-reported medical history of asthma and/or COPD) or asymptomatic allergic state (without CAD or respiratory symptoms such as chronic cough, sputum production, wheezing and dyspnoea).

### 2.4. Other Measurements

Height and weight were measured with a stadiometer and high-precision scale to obtain the waist-height ratio. BMI was additionally calculated as weight/height^2^. Demographics, habits and lifestyles (smoking status, second-hand smoking and cumulative exposures to smoking in pack years), environmental factors (residence type and proximity to a main road) and family history (allergy, COPD and asthma) were obtained via questionnaires. The socio-economic status (SES) score was calculated on a scale of 3–21, and it consisted of the individual’s scale sum of income, education and occupation. Differential cell counts (neutrophils and leukocytes) were additionally measured from blood samples.

Information on symptoms (wheezing, chronic cough, sputum production and dyspnoea) and affirmation of doctor-diagnosed diseases (asthma; COPD including chronic bronchitis and emphysema; cardiovascular disease, CVD; hypertension; allergy; allergic eczema; rhino conjunctivitis; cancer; and neurological disorders) were based on questionnaires and validated according to specific medication use. Participants were assessed for atopy using the skin prick test (for which at least one reaction (≥3 mm) was considered positive). The diagnosis of prediabetes was based on fasting blood glucose ≥ 100–125 mg/dL and/or haemoglobin A1c (HbA1c) values within 5.7–6.4%; diabetes diagnoses were based on fasting blood glucose ≥ 126 mg/dL, HbA1c ≥ 6.5% and/or medication; [16]). Metabolic syndrome was determined by central obesity and at least two of the following: triglycerides ≥ 150 mg/dL or specific medication; high-density lipoprotein cholesterol < 40 mg/dL in men or <50 mg/dL in women, or specific medication; fasting blood glucose ≥100 mg/dL. Hypertension was defined by the following cut-off values: systolic blood pressure ≥ 130 mmHg, diastolic blood pressure ≥ 85 mmHg, or the use of specific medication [17]). Osteopenia and osteoporosis were confirmed via dual-energy X-ray absorptiometry (DXA; Lunar Prodigy, GE Healthcare©, Chicago, IL, USA) scan measurements of the lumbar spine, total hip and neck: osteopenia was determined using a T/Z-score <−1 and ≥−2.5/−2 and osteoporosis via a T/Z-score of <−2.5/−2 [18]. Moderate to advanced kidney disease was considered when the estimated glomerular filtration rate (calculated using the CKD-EPI equation) was <60 mL/min/1.73 m^2^ [19].

### 2.5. Statistical Analyses

All statistical analyses were performed using R version 4.3.0 [20]. Baseline characteristics are expressed as frequency (n [%]), mean ± standard deviation unless stated differently. Comparisons between groups were studied using an analysis of variance (ANOVA) for nominal variables, and chi-squared tests were used for binary variables. Multivariable logistic regressions were used to determine the associations of groups to risk factors, symptoms and comorbidities against the reference group with normal FeNO (<20 ppb) and BEC (<300 cells/µL). Multivariate logistic regressions for the determination of groups to different respiratory subtypes and symptoms were adjusted for age and sex, and a combination of age, sex, BMI, smoking status, cumulative exposures to smoking and familial predisposition to COPD and/or asthma. Pearson’s correlation was used to test associations between BEC and FeNO.

## 3. Results

### 3.1. Prevalence and Clinical Characteristics

**Figure 1:**
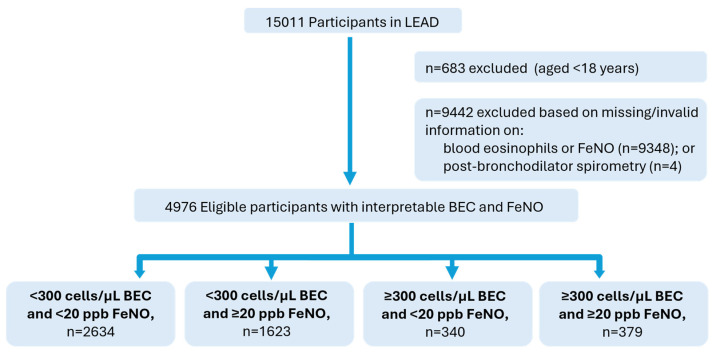
Flowchart of eligible individuals included in the present study.

Among the 4976 participants with valid FeNO, BEC and spirometry measurements in the general population, 2634 (52.9%) individuals had normal FeNO and BEC levels, 1623 (32.6%) had elevated FeNO and normal BEC, 340 (6.8%) with elevated BEC and normal FeNO, and 379 had both elevated FeNO and BEC (7.6%; Figure 1). The characteristics of the participating individuals according to their levels of BEC and FeNO are provided in Table 1. Individuals with elevated FeNO and elevated BEC had the highest proportion of males and lower lung function measurements (in particular, FEV1/FVC, 76.9 ± 8.5% vs. >78% in other groups, *p* < 0.001 for group comparisons), and they were more likely to have respiratory symptoms and asthma. Of notable interest, self-reported allergy was the most common extrapulmonary diagnosis with elevated FeNO in 45.8%, elevated BEC in 35.3% and elevated FeNO with elevated BEC in 53.0%. This was followed by pre- and current diabetes (48.7%), rhino conjunctivitis (46.4%), osteopenia (40.2%) and metabolic syndrome (36.5%) in individuals with elevated FeNO and elevated BEC. Among those with one elevated biomarker, fewer individuals were diagnosed with COPD (only elevated FeNO, 5.9%, and only elevated BEC, 5.0%) and a higher number of individuals diagnosed with asthma (only elevated FeNO, 10.5%, and only elevated BEC, 10.6%, vs. 7.2% in those with FeNO < 20 ppb and BEC < 300 cells/µL). Individuals with elevated FeNO and normal BEC levels were also generally older but shared similarities in low cumulative exposures to smoking, smoking history, anthropometry (BMI and waist–height ratio), leukocyte counts and lung function with individuals with low FeNO and BEC. Individuals with BEC ≥ 300 cells/µL and normal FeNO were younger with the highest values of cumulative exposures to smoking, the proportion of current smokers, BMI, waist–height ratio and white blood cell counts but shared a similar burden of diseases as individuals with only elevated FeNO ≥ 20 ppb or both elevated FeNO and BEC levels. Environmental and living factors and lifestyle were observed to have limited effects on all groups. Individuals with CAD were additionally observed to have lower lung function, reported more symptoms and higher values of inflammatory markers, including FeNO and BEC, compared to individuals without CAD, irrespective of smoking status (Table S1).

**Figure 2:**
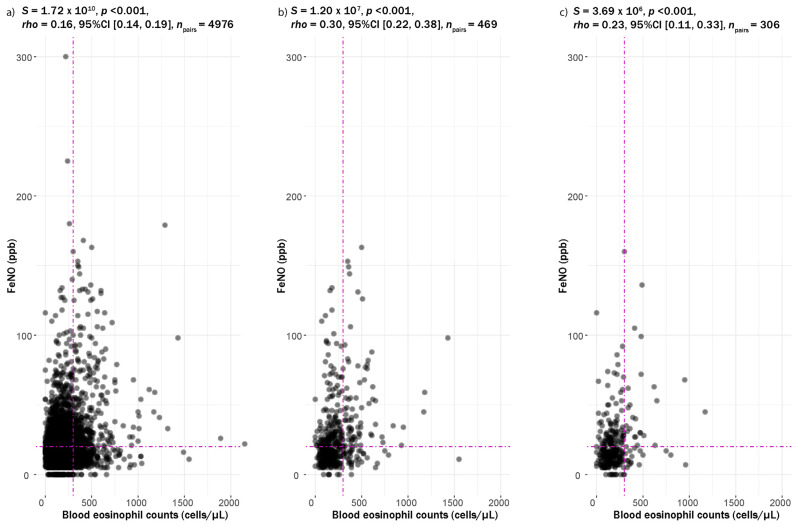
Scatter plot of blood eosinophil counts and FeNO in (**a**) the general population, (**b**) asthmatics and (**c**) individuals with COPD. Correlation analyses are indicated as text above the graph, vertical dotted lines represent the threshold for BEC at 300 cells/µL and horizontal dotted lines represent the threshold for FeNO at 20 ppb.

Correlations of BEC and FeNO were weak but significant (*rho* = 0.16, 95% CI [0.14, 0.19], *p* < 0.001; Figure 2a). Stronger correlations were observed for individuals with asthma (rho = 0.30, *p* < 0.001, Figure 2b) compared to individuals with COPD (*rho* = 0.23, *p* < 0.001; Figure 2c). Correlations between the biomarkers in individuals with CAD were also stronger than those without CAD, regardless of smoking status (Figure S1), and participants who had never smoked were observed to exhibit a stronger correlation between the biomarkers, irrespective of the presence of asymptomatic allergy (Figure S2).

### 3.2. Clinical Attributes

**Figure 3:**
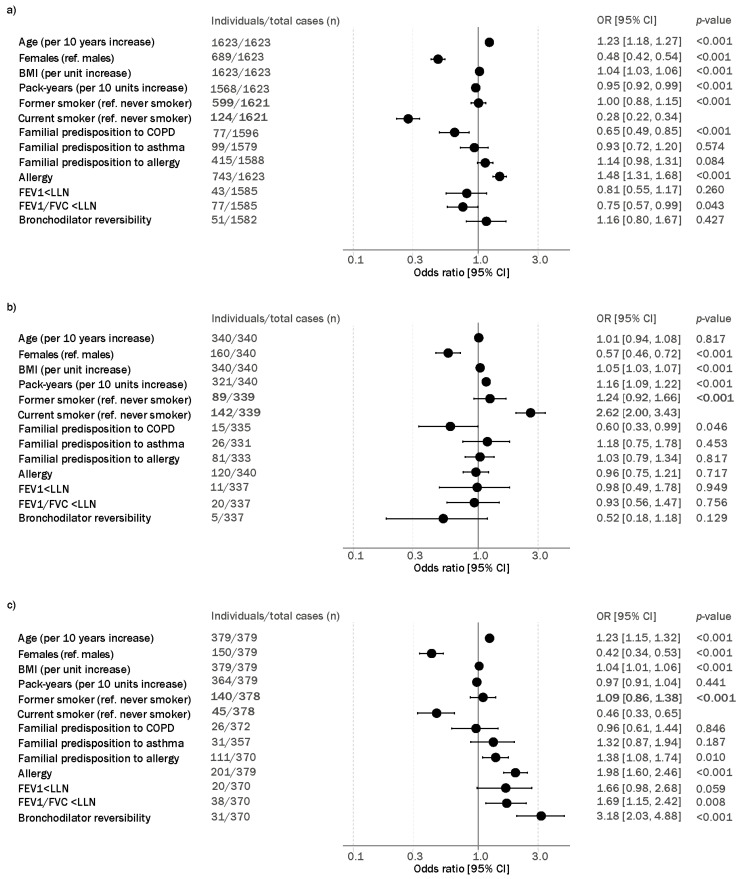
Clinical attributes associated with (**a**) elevated fractional exhaled nitric oxide (FeNO), (**b**) elevated blood eosinophil counts (BEC) and (**c**) T2 inflammation. Individuals are referenced to individuals without elevated FeNO and BEC. Reversibility was defined as forced expiratory volume in 1 s. (FEV1) reversibility of ≥200 mL or ≥12%. Logistic regression models were used, and estimates were unadjusted. *p*-values were obtained from Wald’s test and significance was considered where *p* < 0.05. OR [95% CI], odds ratio [95% confidence intervals]; BMI, body mass index; COPD, chronic obstructive pulmonary disease; FVC, forced vital capacity; and LLN, lower limit of normal.

Univariate regression analysis showed that older age, the male sex, higher values of BMI and former smoking status were associated with an increased risk of having elevated FeNO and BEC both when analysed separately and together (Figure 3). Positive reversibility, allergy, impaired lung function (FEV1/FVC < LLN) and familial predisposition to allergy were significantly associated with the risk of having both elevated FeNO and BEC (OR [95% CI]: 3.18 [2.03, 485], 1.98 [1.60, 2.46], 1.69 [1.15, 2.42] and 1.38 [1.08, 1.74], respectively, *p* ≤ 0.01 for all associations). Higher values of cumulative exposures to smoking were significantly associated with the risk of having only elevated BEC (OR 1.61 [95% CI 1.30, 2.00], *p* < 0.001), whilst allergy was associated with having elevated FeNO levels (OR 1.48 [95% CI 1.31, 1.68]).

### 3.3. Respiratory Symptoms and Chronic Airway Disease

**Figure 4:**
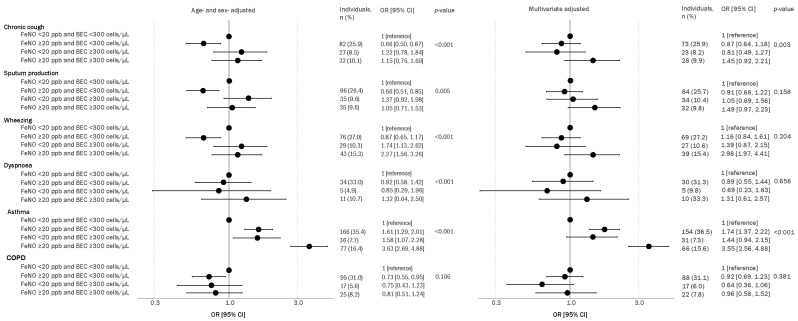
Association of exhaled nitric oxide (FeNO) and blood eosinophil counts (BEC) with respiratory symptoms and diseases. Logistic regression models were used with normal biomarker levels (FeNO < 20 ppb and BEC < 300 cells/µL) as the reference group ([reference]) for all associations and multivariate models were adjusted for age, sex, BMI, smoking status, cumulative exposures to smoking and familial predisposition to chronic obstructive pulmonary disease and asthma. *p*-values were obtained from Wald’s test, and significance was considered where *p* < 0.05. OR [95% CI], odds ratio with 95% confidence intervals.

Chronic cough (OR 1.15 [95% CI 0.76, 1.69]), sputum production (OR 1.05 [95% CI 0.71, 1.53]), wheezing (OR 2.27 [95% CI 1.56, 3.26]) and dyspnoea (OR 1.32 [95% CI 0.64, 2.50]), were associated with individuals with both elevated FeNO and BEC levels, and these associations were maintained after adjustments for potential confounders (Figure 4). Respiratory symptoms were not associated with only elevated FeNO in the age- and sex-adjusted model, whereas only elevated BEC was associated with an increased risk of all respiratory symptoms but not dyspnoea. The corresponding odds ratios (ORs [95% CI]) for elevated FeNO or BEC, respectively, were 0.66 [0.50, 0.87] and 1.22 [0.78, 1.84] for chronic cough, 0.66 [0.51, 0.85] and 1.37 [0.92, 1.98] for sputum production, 0.87 [0.65, 1.17] and 1.74 [1.13, 2.62] for wheezing, and 0.92 [0.58, 1.42] and 0.85 [0.29, 1.96] for dyspnoea. After adjustments for potential confounders, a positive association with wheezing was observed in individuals with only elevated FeNO levels (OR 1.16 [95% CI 0.84, 1.61]), and individuals with only elevated BEC were observed to be less likely to be associated with chronic cough (OR 0.81 [95% CI 0.49, 1.27]).

All three groups were significantly associated with asthma but not COPD (Figure 4). The corresponding odds ratio [95% CI] for asthma in those with either elevated FeNO or BEC was 1.61 [1.29, 2.01]) and 1.58 [1.07, 2.28], respectively, with the greatest association observed in those with elevated FeNO and BEC levels (OR 3.63 [95% CI 2.69, 4.88]; *p* <0.001 for all comparisons). In contrast, all groups were not associated with COPD (OR [95% CI]: only elevated FeNO, 0.73 [0.55, 0.95]; only elevated BEC, 0.75 [0.43, 1.23]; and both elevated FeNO and BEC levels, 0.81 [0.51, 1.24]). Adjustments for additional potential confounders presented similar results. When defining the clinical groups of chronic airway disease using spirometry, the results varied in both the positive reversible test and post-bronchodilator FEV1/FVC < LLN (Figure S3). Individuals with both elevated FeNO and BEC levels were positively associated with reversibility and airflow limitation (sex- and age-adjusted, OR [95% CI]: 2.61 [1.65, 4.03] and 1.36 [0.92, 1.97], respectively; and multivariate-adjusted, OR [95% CI]: 3.03 [1.88, 4.78] and 1.68 [1.08, 2.53], respectively). Elevated BEC but normal FeNO levels are not associated with bronchodilator reversibility, whereas only elevated FeNO is associated with bronchodilator reversibility after adjusting for potential confounders (OR 1.03 [95% CI 0.69, 1.54]). Individuals with one biomarker are not associated with the risk of airflow limitation. Generally, individuals with CAD or healthy individuals with an allergy are more likely to have both elevated biomarkers, irrespective of smoking status (Figure S4).

### 3.4. Extrapulmonary Comorbidities

**Figure 5:**
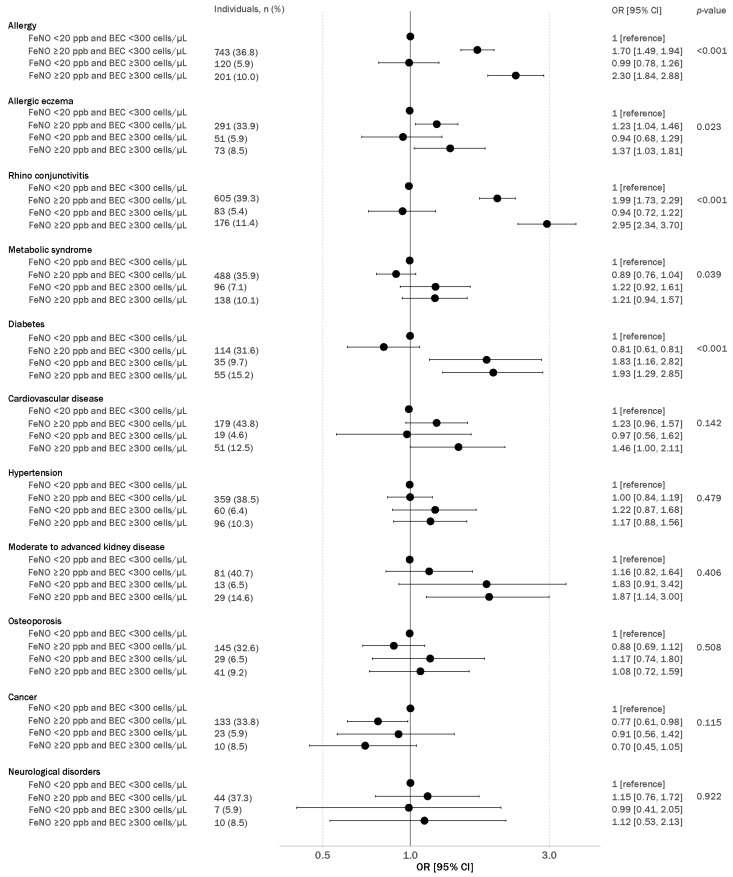
Association of exhaled nitric oxide (FeNO) and blood eosinophil counts (BECs) with extrapulmonary. Logistic regression models were used with normal biomarker levels (FeNO < 20 ppb and BEC < 300 cells/µL) as the reference group ([reference]) for all associations and multivariate models adjusted for age and sex. *p*-values were obtained from Wald’s test, and significance was considered where *p* < 0.05, odds ratio with 95% confidence intervals.

In regression models adjusted for age and sex, elevated FeNO and normal BEC levels, but not elevated BEC or normal FeNO levels, were associated with an increased risk of allergy, allergic eczema and rhino conjunctivitis (OR [95% CI]: 1.70 [1.49, 1.94], 1.23 [1.04, 1.46] and 1.99 [1.73, 2.29], respectively; Figure 5). In contrast, elevated BEC, but not elevated FeNO, was associated with metabolic syndrome and diabetes (OR [95% CI]: 1.22 [0.92, 1.61] and 1.83 [1.16, 2.82], respectively). The strongest associations in the abovementioned comorbidities were found in individuals with both elevated FeNO and BEC levels with corresponding odds ratios of 2.30 [1.84, 2.88] for allergy, 1.37 [1.03, 1.81] for allergic eczema, 2.95 [2.3, 3.70] for rhino conjunctivitis, 1.80 [1.26, 2.54] for diabetes and 1.21 [0.94, 1.57] for metabolic syndrome. Although osteoporosis, kidney disease, hypertension, cardiovascular disease and neurological disorders presented associations with both elevated FeNO levels and BEC, these associations did not reach statistical significance. Collectively, elevated BEC only is sufficient for associations with respiratory symptoms or diagnosed respiratory diseases, whilst elevated FeNO only is more sensitive for associations with extrapulmonary conditions, with an additive effect with a combination of elevated FeNO and BEC (Figure S5).

## 4. Discussion

This study shows that screening for elevated markers of T2 inflammation in the general population offers the following observations: (1) the combined increase in FeNO and BEC has a high potential to predict respiratory pathologies associated with asthma and allergic diseases; (2) elevated levels of one of the biomarkers can discriminate between different risk associations of FeNO and BEC: FeNO is highly sensitive to asthma and atopic states but is not predictive of respiratory symptoms or airflow obstruction; and BEC is associated very strongly with asthma, airflow obstruction and bronchial hyperreactivity, as well as smoking exposure and systemic metabolic diseases; and (3) biomarkers of T2 inflammation are not associated with diagnosed COPD or any other comorbidity profile aside from metabolic diseases. The strong association of the biomarkers to asthma and its different clinical features might be useful in detecting unrecognised asthma and supporting the differentiation between asthma and COPD in fixed airflow obstruction.

In an unbiased analysis of the general population, the prevalence of an elevated FeNO with normal BEC was 32.6%, elevated BEC with normal FeNO was 6.8% and elevated FeNO and BEC was 7.6%. This discordancy in FeNO levels and BEC is similarly observed in previous reports [21,22]. The high prevalence of individuals with both elevated biomarkers cannot be solely explained by the association with asthma, for which FeNO is used as an indirect measure of T2 inflammation triggered via allergens [20]. This pathway is also associated with other atopic states, and it was confirmed in our analyses: single FeNO elevation was associated with eczema, allergic rhinosinusitis, hay fever and asthma. On the other hand, the single elevation of BEC ≥ 300 cells/µL was associated not only with asthma but also with cumulative and current obstructive lung function. However, the discordancy in associations of BEC and FeNO presented in this study suggests that the use of both T2 biomarkers could be clinically useful in the assessment of different airway inflammation for the identification of patients suitable for different biologics. A prime example of this is anti-IL5 treatments with mepolizumab greatly reducing BEC with negligible effects on FeNO [6,23] and, in contrast, anti-IL13 (lebrizumab) and anti-IL4 and −13 (dupilumab) treatments reducing FeNO without notable changes in BEC [3,24].

Although the FeNO and BEC combination is commonly thought to measure eosinophilic airway inflammation [14], correlations between FeNO and (blood or sputum) eosinophils have consistently been reported to be weak [4,21]. In this study, we also reported a weak correlation, albeit significant (*rho* = 0.16, *p* <0.001), for correlations between FeNO and BEC in the general population and a stronger correlation between these biomarkers in respiratory conditions. An additive effect of the combined elevated FeNO and BEC levels was found in this study to present higher odds ratios and associations with respiratory and extrapulmonary conditions compared to when only one elevated biomarker was studied. Indeed, the additive associations of FeNO and BEC levels can be speculated to result from different regulations of the biomarkers via the activation of different cytokine mechanisms with eosinophils as systemic and FeNO as local T2 markers, resulting in a “double-hit” mechanism for the development respiratory symptoms, asthma and airflow limitation [25]. Although a combination of elevated FeNO and BEC was strongly related to respiratory conditions, it should be noted that a significant proportion of individuals reported neither. It is possible that asymptomatic subjects with a combination of elevated FeNO and BEC constitute a group of individuals with a high risk of developing obstructive lung diseases or symptoms in the future.

Regression analyses in this study also showed a weaker association with asthma in individuals with elevated BEC only, despite the findings of BEC being independently associated with respiratory symptoms aligned with previous findings [4,26]. Interestingly individuals with both elevated biomarkers were shown to have lower lung function despite the proportion of individuals with COPD diagnosis being considerably low (6.6%) in this study. This was similarly observed by Çolak and colleagues, who reported in their study that elevated FeNO and BEC were jointly associated with asthma and asthma–COPD overlap (ACO) but not with COPD [8]. They suggested that FeNO and BEC are unlikely to differentiate asthma from COPD and instead identify pathophysiological mechanisms more common to asthma and ACO than COPD [8,27]. However, it must be noted that individuals with both elevated biomarkers were associated with airflow limitation and bronchial reactivity but not COPD diagnosis in the regression analyses [8]. Despite not having an independent ACO group in this study, both biomarkers were still observed to have discriminated between features of asthma phenotypes and thereby fulfilled the prerequisites for asthma treatment. The findings in this study, however, warrant further interest in screening for both biomarkers of T2 inflammation in the clinic to predict and potentially diagnose more precisely the underlying phenotype.

The pleiotropism of BEC and FeNO is also worth discussing. This study found a significant independent association between extrapulmonary diseases and elevated BEC only and not FeNO. High BEC is also known to be an independent prognostic and long-term mortality factor in cardiovascular and cerebrovascular events in triple-vessel coronary artery disease [28,29] and an increased risk for chronic kidney progression [30,31,32,33]. Heart disease, stroke and/or metabolic disease have been previously shown to be independently associated with BEC but not FeNO [34]. Lower FeNO is also known to be a pathophysiological mediator of hypertension [35], which aligned with our findings. Previously, decreased FeNO levels in patients with pulmonary hypertension were attributed to alterations in levels or activity of NOS enzymes (types I, II and III), known to endogenously synthesise and thereby regulate blood-vessel tone, resulting in decreased NO reaction products and NO levels in the lungs of patients with pulmonary hypertension [14,36,37]. The associations of T2-biomarkers explored in this study demonstrate that monitoring FeNO and BEC levels over time is an effective and non-invasive maker to evaluate not only pulmonary but also extrapulmonary conditions in patients [38].

Our study exhibited several strengths that are worth noting: FeNO and BEC were jointly assessed in the general population, and we have presented findings of the characteristics and prevalence of T2 biomarkers in which FeNO is poorly studied; moreover, the validity of measuring FeNO and BEC were further assessed according to their influence on pulmonary and extrapulmonary conditions. Among potential limitations, we acknowledge that the definition of clinical groups of allergy, CAD, symptoms and other diseases was dependent on affirmative self-reported answers and may, therefore, have resulted in the misclassification of individuals. We have accordingly provided additional definitions based on lung function derived from spirometry, as well as skin prick tests, to reduce the likelihood of the misclassification of individuals with respiratory diseases and atopy, respectively.

These findings also cannot conclusively inform readers about causal effects of translatability to diseased cohorts. We have nevertheless determined that the combination of elevated FeNO and BEC had a contributory effect in most pulmonary and extrapulmonary conditions. Additionally, although BEC is a known surrogate biomarker for sputum eosinophils, there remains a poor concordance between both biomarkers in the literature, especially in the reflection on localised airway inflammation and the clinical utility in airflow obstruction [27,39]. Arguably, obtaining BEC is less invasive, more accessible and requires a lower technical difficulty, particularly since this current study has established the importance of BEC in routine clinical testing. Whilst BEC may be influenced by other therapies, disease conditions or systemic inflammation, and may thereby reduce specificity, BEC still offers good predictive value for corticosteroid responsiveness and biologic therapies relative to sputum eosinophils [40,41].

## 5. Conclusions

This present study affirms that elevated FeNO levels or BEC can identify potential respiratory symptoms and diagnoses in the general population. The combination of both factors presents synergistic effects in discerning between these conditions, particularly respiratory symptoms and asthma, but not COPD, and this combination extends to extrapulmonary conditions.

## Figures and Tables

**Table 1:** Cross-sectional analysis of demographics, biomarkers, and clinical characteristics of individuals with various levels of Type-2 biomarkers.

	FeNO < 20 ppb and BEC < 300 cells/µL	FeNO ≥ 20 ppb and BEC < 300 cells/µL	FeNO < 20 ppb and BEC ≥ 300 cells/µL	FeNO ≥ 20 ppb and BEC ≥ 300 cells/µL	*p*-Value
Total n (%)	2634 (52.9)	1623 (32.6)	340 (6.8)	379 (7.6)	
Males	1034 (39.3)	934 (57.5)	180 (52.9)	229 (60.4)	<0.001
Age, years	50.1 ± 16.3	55.5 ± 16.2	50.3 ± 14.5	55.6 ± 16.8	<0.001
BMI, kg/m^2^	25.9 ± 4.8	26.7 ± 4.5	27.1 ± 5.0	26.5 ± 4.7	<0.001
Waist–height ratio	0.561 ± 0.1	0.571 ± 0.1	0.573 ± 0.1	0.571 ± 0.1	<0.001
**Exposures and lifestyle**					
Pack years	9.1 ± 16.7	7.9 ± 16.3	14.6 ± 19.4	8.4 ± 16.8	<0.001
Smoking status					
Never	1215 (46.2)	898 (55.4)	108 (31.9)	193 (51.1)	<0.001
Former	807 (30.7)	599 (37.0)	89 (26.3)	140 (37.0)	
Current	610 (23.2)	124 (7.6)	142 (41.9)	45 (11.9)	
Socio-economic score	13.7 ± 2.9	14.1 ± 2.8	13.5 ± 3.0	13.8 ± 2.9	<0.001
Low income (<€1100/month)	235 (9.0)	97 (6.1)	34 (10.1)	27 (7.3)	0.003
Low education	738 (28.1)	421 (26.0)	113 (33.3)	104 (27.6)	0.047
Rural residence	447 (17.2)	273 (17.0)	59 (17.5)	63 (16.8)	0.994
Living on or near a main road	1490 (56.6)	847 (52.3)	186 (54.9)	211 (55.8)	0.048
Dust exposure	545 (20.7)	342 (21.1)	82 (24.2)	82 (21.8)	0.520
Familial predisposition to COPD	188 (7.3)	77 (4.8)	15 (4.5)	26 (7.0)	0.007
Familial predisposition to asthma	171 (6.7)	99 (6.3)	26 (7.9)	31 (8.7)	0.352
Familial predisposition to allergy	613 (23.8)	415 (26.1)	81 (24.3)	111 (30.0)	0.041
**Inflammatory biomarkers**					
FeNO, ppb	12.1 ± 4.2	31.4 ± 17.0	11.8 ± 4.3	45.2 ± 31.4	<0.001
Eosinophil, cells/µL	133.1 ± 67.5	148.8 ± 70.6	431.4 ± 162.0	459.7 ± 212.5	<0.001
Neutrophils, cells/L	4.0 ± 1.4	3.9 ± 1.4	4.3 ± 1.5	4.0 ± 1.4	<0.001
Leukocytes, cells/L	6.8 ± 1.8	6.6 ± 1.8	7.9 ± 2.0	7.4 ± 1.7	<0.001
**Pulmonary measurements**					
FEV1, % predicted GLI	102.8 ± 14.1	103.5 ± 14.6	100.8 ± 13.1	99.9 ± 15.8	<0.001
FVC, % predicted GLI	104.0 ± 13.2	104.1 ± 13.7	102.9 ± 12.5	102.6 ± 14.1	0.110
FEV1, L	3.3 ± 0.9	3.4 ± 0.9	3.4 ± 0.8	3.3 ± 1.0	0.005
FVC, L	4.2 ± 1.0	4.3 ± 1.1	4.3 ± 1.0	4.3 ± 1.1	<0.001
FEV1/FVC, %	79.3 ± 7.8	78.4 ± 7.1	78.3 ± 7.3	76.9 ± 8.5	<0.001
**Respiratory symptoms**					
Sputum production	197 (7.5)	96 (5.9)	35 (10.3)	35 (9.2)	0.010
Wheezing	133 (5.0)	76 (4.7)	29 (8.5)	43 (11.3)	<0.001
Dyspnoea	53 (2.0)	34 (2.1)	5 (1.5)	11 (2.9)	0.584
Chronic cough	175 (6.6)	82 (5.1)	27 (7.9)	32 (8.4)	0.027
**Respiratory diagnosis**					
Positive reversible test	72 (2.8)	51 (3.2)	5 (1.5)	31 (8.4)	<0.001
Asthma	190 (7.2)	166 (10.2)	36 (10.6)	77 (20.3)	<0.001
COPD	169 (6.4)	95 (5.9)	17 (5.0)	25 (6.6)	0.687
Post-bronchodilator FEV1/FVC < LLN	164 (6.4)	77 (4.9)	20 (5.9)	38 (10.3)	0.001
Post-bronchodilator FEV1 <LLN	86 (3.3)	43 (2.7)	11 (3.3)	20 (5.4)	0.075
**Other clinical characteristics**					
Positive skin prick test	1056 (41.8)	831 (53.2)	131 (41.5)	208 (59.6)	<0.001
Allergy	956 (36.3)	743 (45.8)	120 (35.3)	201 (53.0)	<0.001
Allergic eczema	444 (16.9)	291 (17.9)	51 (15.0)	73 (19.3)	0.378
Rhino conjunctivitis	675 (25.6)	605 (37.3)	83 (24.4)	176 (46.4)	<0.001
Cardiovascular disease	160 (6.1)	179 (11.0)	19 (5.6)	51 (13.5)	<0.001
Hypertension	418 (15.9)	359 (22.1)	60 (17.6)	96 (25.3)	<0.001
Moderate kidney disease	76 (2.9)	81 (5.0)	13 (3.8)	29 (7.7)	<0.001
eGFR, ml/min/1.73 m^2^	94.6 ± 17.4	89.6 ± 17.7	93.7 ± 17.7	89.3 ± 19.8	<0.001
Osteopenia	1000 (38.0)	669 (41.2)	139 (40.9)	145 (38.3)	0.301
Osteoporosis	230 (8.7)	145 (8.9)	29 (8.5)	41 (10.8)	
Metabolic syndrome	638 (24.3)	488 (30.1)	96 (28.3)	138 (36.5)	<0.001
Prediabetes	820 (31.3)	629 (38.9)	105 (31.1)	129 (34.1)	<0.001
Diabetes	157 (6.0)	114 (7.1)	35 (10.4)	55 (14.6)	
Neurological disorders	57 (2.2)	44 (2.7)	7 (2.1)	10 (2.6)	0.669

Data are presented as frequency (n [%]) or mean ± standard deviation. FeNO, fractional exhaled nitric oxide; BEC, blood eosinophil count; BMI, body mass index; COPD, chronic obstructive pulmonary disease; FEV1, forced expiratory volume in 1 second; GLI, Global Lung Initiative; FVC, forced vital capacity; LLN, lower limit of normal; and eGFR, estimated glomerular filtration rate. Significance is considered to be *p* < 0.05.

## Data Availability

The data presented in this study are available upon request from the corresponding author due to privacy concerns.

## Supplementary Materials

The following supporting information can be downloaded at https://www.mdpi.com/article/10.3390/jcm13226662/s1: Table S1: Characteristics of individuals stratified according to smoking status and chronic airway disease. Figure S1: Correlations between fractional exhaled nitric oxide (FeNO) and blood eosinophil counts in never- and ever-smokers with and without with and without self-reported medical history of chronic airway disease (CAD; asthma and/or chronic obstructive pulmonary disease). Horizontal lines represent the FeNO threshold of 20 ppb, and vertical lines represent the blood eosinophil count threshold of 300 cells/µL. Figure S2: Correlations between fractional exhaled nitric oxide (FeNO) and blood eosinophil counts in healthy never- and ever-smokers (without self-reported medical history of asthma and/or chronic obstructive pulmonary disease nor respiratory symptoms). Horizontal lines represent the FeNO threshold of 20 ppb, and vertical lines represent the blood eosinophil count threshold of 300 cells/µL. Figure S3: Association between exhaled nitric oxide (FeNO) and blood eosinophil counts (BEC) with respiratory subtypes defined via spirometry Logistic regression models were used with normal biomarker levels (FeNO < 20 ppb and BEC < 300 cells/µL) as the reference group ([reference]) for all associations and multivariate models were adjusted for age, sex, BMI, smoking status, cumulative exposures to smoking and familial predisposition to chronic obstructive pulmonary disease and asthma. *p*-values were obtained from Wald’s test, and significance was considered where *p* <0.05. OR [95% CI], odds ratio with 95% confidence intervals. Figure S4: Odd ratios of elevated fractional exhaled nitric oxide (FeNO) levels and/or blood eosinophil counts (BECs) in never- and ever-smokers with chronic airway disease (CAD) or those who are healthy (without CAD and respiratory symptoms) but have an allergy. Logistic regression models were used with normal biomarker levels (FeNO < 20 ppb and BEC < 300 cells/µL) as the reference group for all associations and are adjusted for age and sex. Estimates are odds ratios (ORs [95% confidence intervals]) for which significance was considered where *p* < 0.05. Figure S5: Odds ratios for elevated fractional exhaled nitric oxide (FeNO) levels and/or blood eosinophil counts (BECs) presenting respiratory and extrapulmonary conditions Logistic regression models were used with normal biomarker levels (FeNO < 20 ppb and BEC < 300 cells/µL) as the reference group for all association and are adjusted for age and sex. Estimates are odds ratios (ORs [95% confidence intervals]) for which significance was considered where *p* < 0.05.
