# Supplementary material for: Type-2 Inflammation in Health and Disease: Prevalence, Risk Factors and Multimorbidity
Source: J Clin Med. 2024 Nov 6;13(22):6662. doi: 10.3390/jcm13226662 (PMC11594687; doi:10.3390/jcm13226662)
Supplement: Supplementary file 1 [file jcm-13-06662-s001.zip › jcm-3287080-supplementary.pdf]

Type-2 Inflammation in Health and Disease: Prevalence, Risk Factors and Multimorbidity

Charmaine J. M. Lim [1,2], Christoph Gross [1,2], Marie-Kathrin Breyer [1,3], Robab Breyer-Kohansal [1,4], Emiel F. M. Wouters [1,2,5,*] and Sylvia Hartl [1,2]

1 Ludwig Boltzmann Institute for Lung Health, 1140 Vienna, Austria;
2 Faculty of Medicine, Sigmund Freud Private University, 1020 Vienna, Austria
3 Department of Respiratory and Pulmonary Diseases, Clinic Penzing, Vienna Healthcare Group, 1140 Vienna, Austria
4 Department of Respiratory and Pulmonary Diseases, Clinic Hietzing, Vienna Healthcare Group, 1130 Vienna, Austria
5 Nutrition and Translational Research in Metabolism (NUTRIM), Maastricht University Medical Center, 6229 ER Maastricht, The Netherlands
* Correspondence: woutersemiel@gmail.com

Supplementary Materials

Table S1 Characteristics of individuals stratified according to smoking status and chronic airway disease.

	Never smokers			Ever-smokers		
	No CAD	CAD	Total	No CAD	CAD	Total
Total n (%)	2128 (49.9)	286 (40.7)	2414 (48.6)	2140 (50.1)	416 (59.3)	2556 (51.4)
Males	936 (44.0)	127 (44.4)	1063 (44.0)	1103 (51.5)*	208 (50.0)*	1311 (51.3)*
Age, years	50.7 ± 17.8	52.1 ± 18.1	50.9 ± 17.8	53.3 ± 14.9*	55.0 ± 14.6*	53.6 ± 14.8*
BMI, kg/m^2	25.7 ± 4.7	26.3 ± 4.8	25.8 ± 4.7	26.5 ± 4.7*	27.4 ± 5.2*	26.7 ± 4.8*
**Lifestyles and exposures**						
Pack-years	NA	NA	NA	17.0 ± 19.3	23.9 ± 23.3	18.1 ± 20.2
Current smokers	NA	NA	NA	758 (35.4)	163 (39.2)	921 (36.0)
Dust exposure	14.0 (3.0)	14.0 (3.0)	433 (17.9)	13.8 (2.7)*	13.3 (2.8)*	618 (24.2)*
Familial predisposition to COPD	167 (8.0)	26 (9.2)	123 (5.2)	160 (7.5)*	40 (9.9)*	183 (7.3)*
Familial predisposition to asthma	461 (21.7)	75 (26.2)	160 (6.9)	674 (31.5)	166 (39.9)	167 (6.7)
Familial predisposition to allergy	1726 (81.9)	238 (84.1)	634 (26.9)	1769 (83.6)*	347 (83.6)*	586 (23.3)*
Second-hand smoking	1128 (53.0)	175 (61.2)	675 (28.0)	1187 (55.5)*	244 (58.7)*	1162 (45.5)*
Parental smoking	375 (17.6)	58 (20.3)	1249 (52.2)	502 (23.5)*	116 (27.9)*	1487 (59.1)*
Socio-economic score	99 ± 4.7	24 ± 8.5	14.0 ± 3.0	140 ± 6.7*	43 ± 10.6*	13.7 ± 2.8*
Low income (<£1100/month)	120 (5.8)	40 (14.8)	193 (8.1)	128 (6.2)	39 (9.7)	200 (7.9)
Low education	535 (25.7)	99 (35.6)	536 (22.2)	473 (22.5)*	113 (27.5)*	840 (32.9)*
Urban residence	588 (27.6)	87 (30.4)	1964 (82.2)	943 (44.1)	219 (52.6)	2116 (83.6)
Living on or near a main road	1091 (51.7)	158 (55.6)	1303 (54.0)	1237 (58.5)	250 (61.7)	1431 (56.0)
**Lung function**						
FEV1, %predicted GLI	104.7 ± 13.3	96.7 ± 15.9	103.8 ± 13.9	103.5 ± 13.3*	92.0 ± 17.7*	101.6 ± 14.7*
FVC, %predicted GLI	103.9 ± 13.5	102.0 ± 13.7	103.7 ± 13.5	104.5 ± 13.0	101.9 ± 14.5	104.0 ± 13.3
FEV1, L	3.4 ± 0.9	3.1 ± 0.9	3.4 ± 0.9	3.4 ± 0.8	2.9 ± 0.9	3.3 ± 0.9*
FVC, L	4.2 ± 1.1	4.0 ± 1.1	4.2 ± 1.1	4.3 ± 1.0*	4.1 ± 1.1	4.3 ± 1.0
FEV1/FVC, %	80.7 ± 6.5	75.7 ± 9.1	80.1 ± 7.1	78.7 ± 6.6*	71.4 ± 10.8*	77.5 ± 7.9*
**Clinical characteristics**						
Positive reversibility	48 (2.3)	25 (9.0)	73 (3.1)	42 (2.0)	44 (10.8)	86 (3.5)
FEV1 <LLN	819 (38.5)	199 (69.6)	57 (2.4)	774 (36.2)	228 (54.8)	103 (4.1)*
FEV1/FVC <LLN	36 (1.7)	21 (7.5)	94 (4.0)	44 (2.1)*	59 (14.4)*	204 (8.2)*
Sputum production	60 (2.8)	46 (16.1)	106 (4.4)	167 (7.8)*	90 (21.6)*	257 (10.1)*
Wheezing	38 (1.8)	57 (19.9)	95 (3.9)	92 (4.3)*	94 (22.6)*	186 (7.3)*
Dyspnoea	29 (1.4)	18 (6.3)	47 (1.9)	20 (0.9)	36 (8.7)	56 (2.2)
Chronic cough	71 (3.3)	35 (12.2)	106 (4.4)	129 (6.0)*	81 (19.5)*	210 (8.2)*

Positive skin prick test	967 (46.9)	175 (70.9)	1082 (47.3)	877 (42.4) *	205 (56.0)*	1142 (42.3)*
Allergy	50 (2.4)	44 (15.7)	1018 (42.2)	85 (4.1)	119 (29.1)	1002 (39.2)*
**Inflammatory biomarkers**						
FeNO, ppb	21.4 ± 16.5	31.8 ± 27.8	22.6 ± 18.5	18.5 ± 14.4*	22.1 ± 21.8*	19.1 ± 15.9*
Eosinophil, cells/μL	165.7 ± 131.5	222.9 ± 190.5	172.5 ± 141.0	190.0 ± 145.8*	212.9 ± 156.2	193.7 ± 147.8*
Neutrophils, cells/L	3.8 ± 1.3	3.8 ± 1.3	3.8 ± 1.3	4.1 ± 1.5*	4.3 ± 1.7*	4.1 ± 1.5*
Leukocytes, cells/L	6.6 ± 1.7	6.7 ± 1.7	6.6 ± 1.7	7.0 ± 1.9*	7.4 ± 2.1*	7.1 ± 1.9*

Data is presented as frequency (n [%]) or mean ± standard deviation. CAD, chronic airway disease (self-reported medical history of asthma and/or COPD); BEC, blood eosinophil count; BMI, body mass index; COPD, chronic obstructive pulmonary disease; FEV1, forced expiratory volume in 1s; GLI, Global Lung Initiative; FVC, forced vital capacity; LLN, lower limit of normal; and FeNO, fractional exhaled nitric oxide. Significance is considered where $p<0.05$: *statistical significance for comparisons to never-smokers.

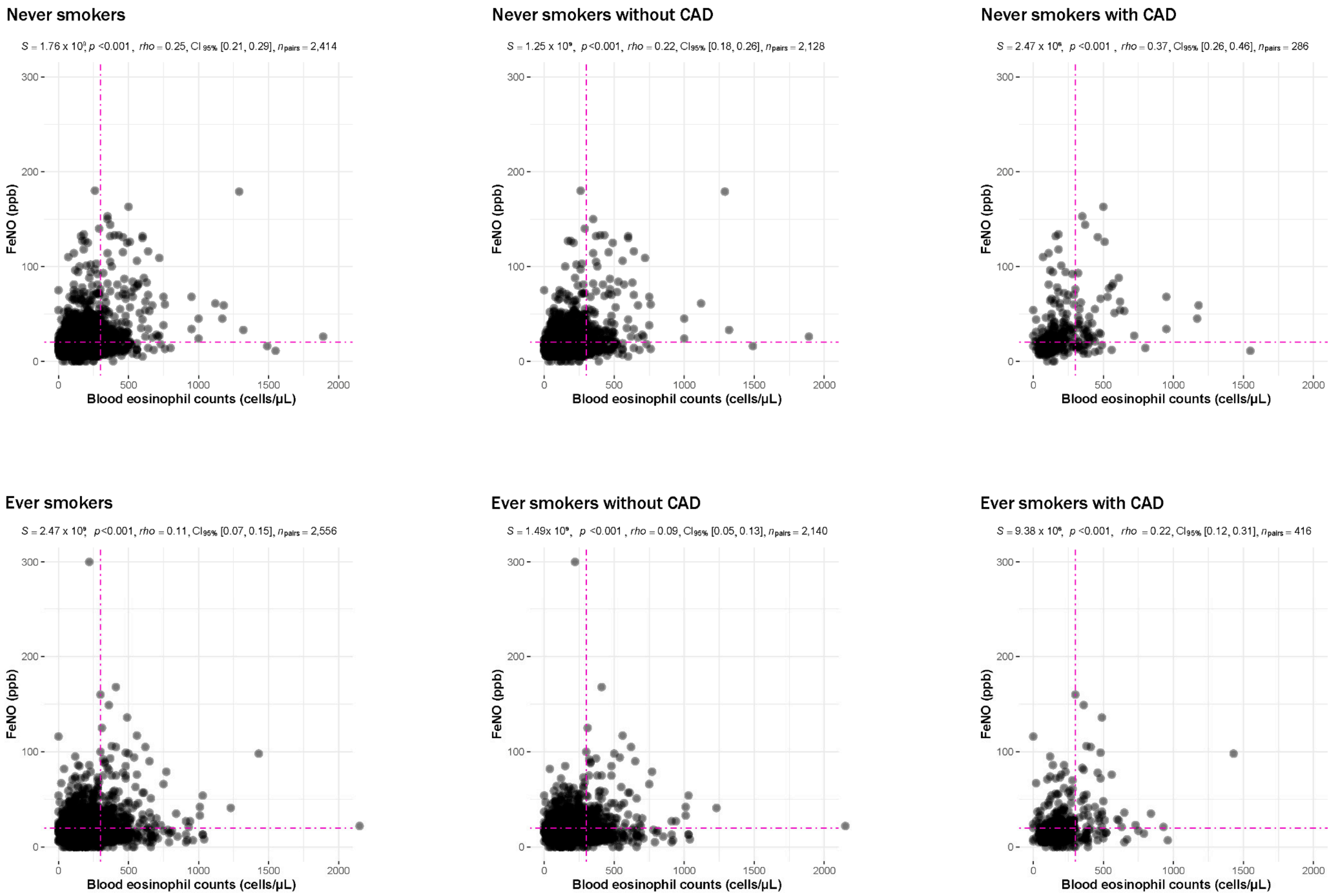

Never smokers
S = 1.76 x 10⁹, p <0.001 , rho = 0.25, CI 95% [0.21, 0.29], n pairs = 2,414
FeNO (ppb)
Blood eosinophil counts (cells/μL)
Never smokers without CAD
S = 1.25 x 10⁹, p <0.001, rho = 0.22, CI95% [0.18, 0.26], n pairs = 2,128
FeNO (ppb)
Blood eosinophil counts (cells/μL)
Never smokers with CAD
S = 2.47 x 10⁶, p <0.001 , rho = 0.37, CI95% [0.26, 0.46], n pairs = 286
FeNO (ppb)
Blood eosinophil counts (cells/μL)
Ever smokers
S = 2.47 x 10⁹, p <0.001, rho = 0.11, CI95% [0.07, 0.15], n pairs = 2,556
FeNO (ppb)
Blood eosinophil counts (cells/μL)
Ever smokers without CAD
S = 1.49x 10⁹, p <0.001 , rho = 0.09, CI95% [0.05, 0.13], n pairs = 2,140
FeNO (ppb)
Blood eosinophil counts (cells/μL)
Ever smokers with CAD
S = 9.38 x 10⁶, p <0.001, rho = 0.22, CI95% [0.12, 0.31], n pairs = 416
FeNO (ppb)
Blood eosinophil counts (cells/μL)

Figure S1 Correlations of fractional exhaled nitric oxide (FeNO) and blood eosinophil counts in never- and ever-smokers with and without self-reported medical history of chronic airway disease (CAD; asthma and/or chronic obstructive pulmonary disease). Horizontal lines represent the FeNO threshold of 20 ppb and vertical lines represent the blood eosinophil count threshold of 300 cells/μL.

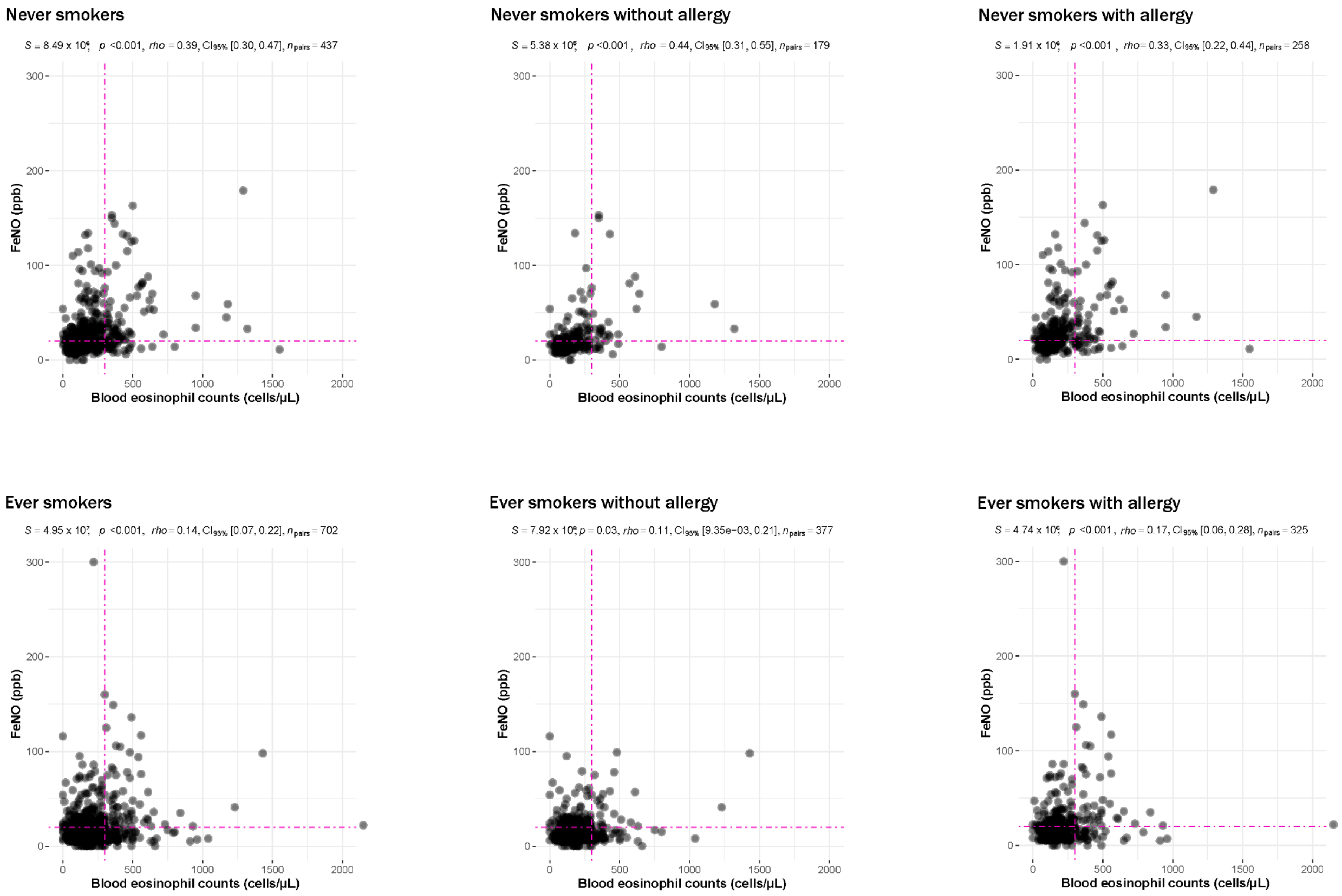
Never smokers
S = 8.49 x 10^6, p <0.001, rho = 0.39, CI95% [0.30, 0.47], n pairs = 437
Never smokers without allergy
S = 5.38 x 10^5, p <0.001, rho = 0.44, CI95% [0.31, 0.55], n pairs = 179
Never smokers with allergy
S = 1.91 x 10^6, p <0.001, rho = 0.33, CI95% [0.22, 0.44], n pairs = 258
Ever smokers
S = 4.95 x 10^7, p <0.001, rho = 0.14, CI95% [0.07, 0.22], n pairs = 702
Ever smokers without allergy
S = 7.92 x 10^6, p = 0.03, rho = 0.11, CI95% [9.35e−03, 0.21], n pairs = 377
Ever smokers with allergy
S = 4.74 x 10^6, p <0.001, rho = 0.17, CI95% [0.06, 0.28], n pairs = 325
FeNO (ppb)
Blood eosinophil counts (cells/µL)
0
100
200
300
500
1000
1500
2000

Figure S2 Correlations of fractional exhaled nitric oxide (FeNO) and blood eosinophil counts in never- and ever-smokers in healthy (without self-reported medical history of asthma and/or chronic obstructive pulmonary disease nor respiratory symptoms). Horizontal lines represent the FeNO threshold of 20 ppb and vertical lines represent the blood eosinophil count threshold of 300 cells/μL.

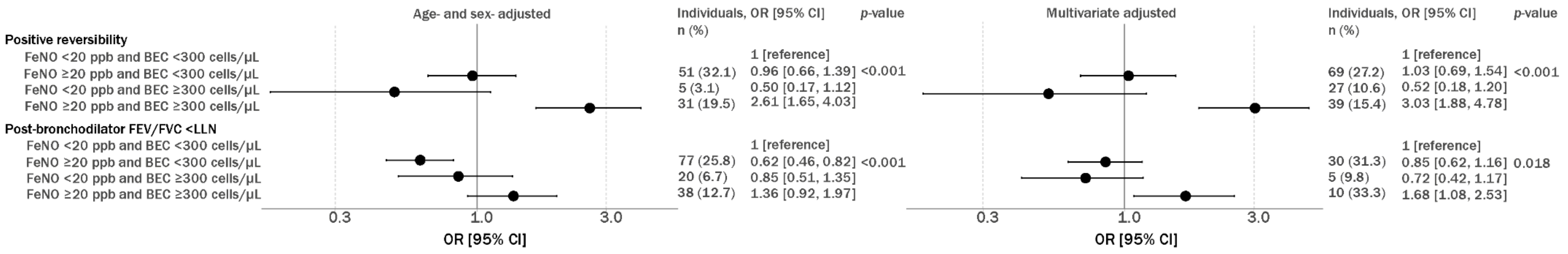

Figure S3 Association of exhaled nitric oxide (FeNO) and blood eosinophil counts (BEC) with respiratory subtypes defined by spirometry. Logistic regression models were used with normal biomarker levels (FeNO <20 ppb and BEC <300 cells/µL) as the reference group ([reference]) for all associations and multivariate models were adjusted for age, sex, BMI, smoking status, cumulative exposures to smoking and familial predisposition to chronic obstructive pulmonary disease and asthma. *p*-values were obtained from Wald's test and significance was considered where *p* <0.05. OR [95% CI], odds ratio with 95% confidence intervals.

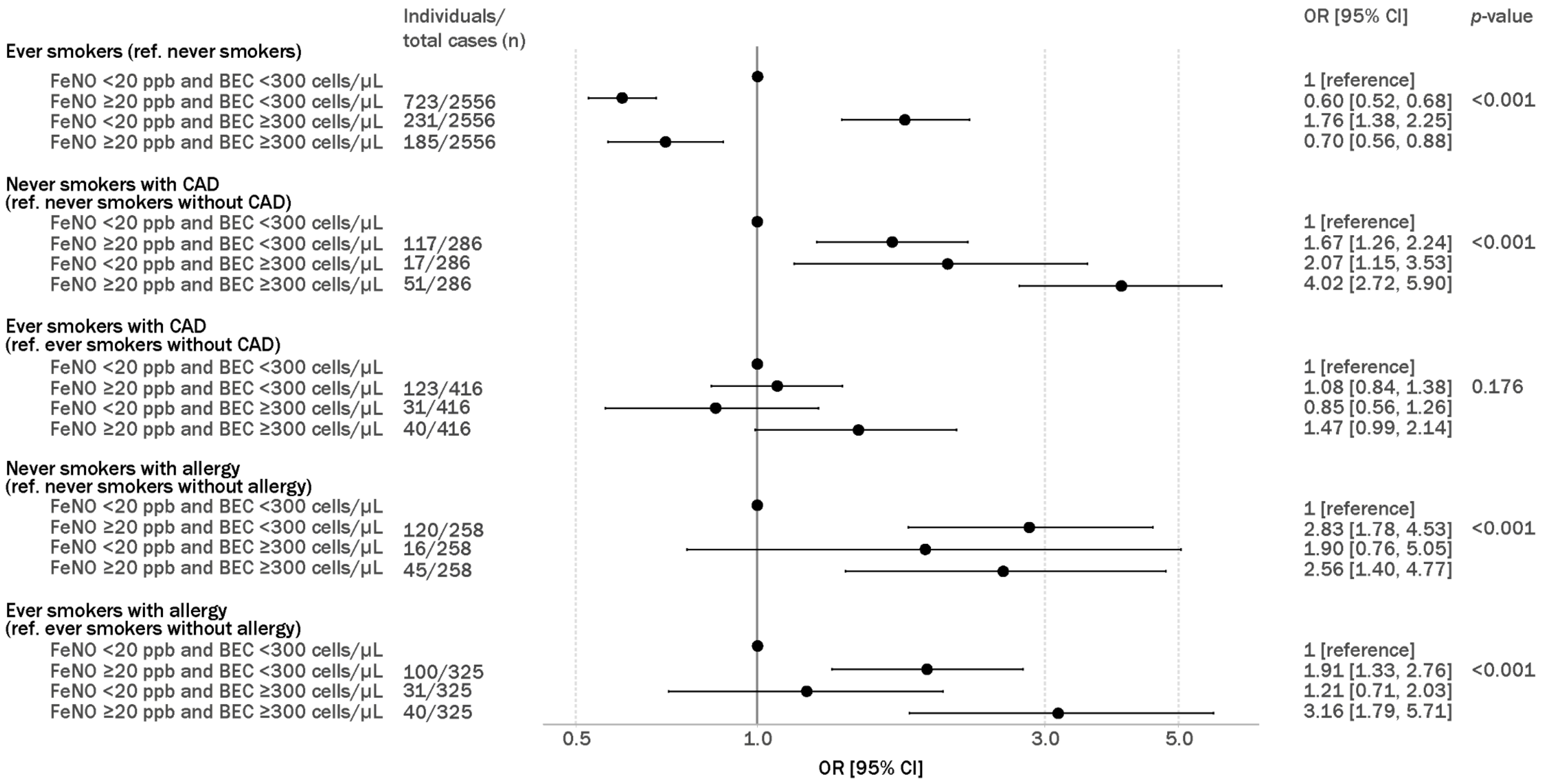

Figure S4 Odd ratios of elevated fractional exhaled nitric oxide (FeNO) levels and/or blood eosinophil counts (BEC) in never- and ever-smokers with chronic airway disease (CAD) or those who are healthy (without CAD and respiratory symptoms) but have an allergy. Logistic regression models were used with normal biomarker levels (FeNO <20 ppb and BEC <300 cells/µL) as the reference group for all associations and are adjusted for age and sex. Estimates are odds ratios (OR [95% confidence intervals]) where significance was considered where *p* <0.05.

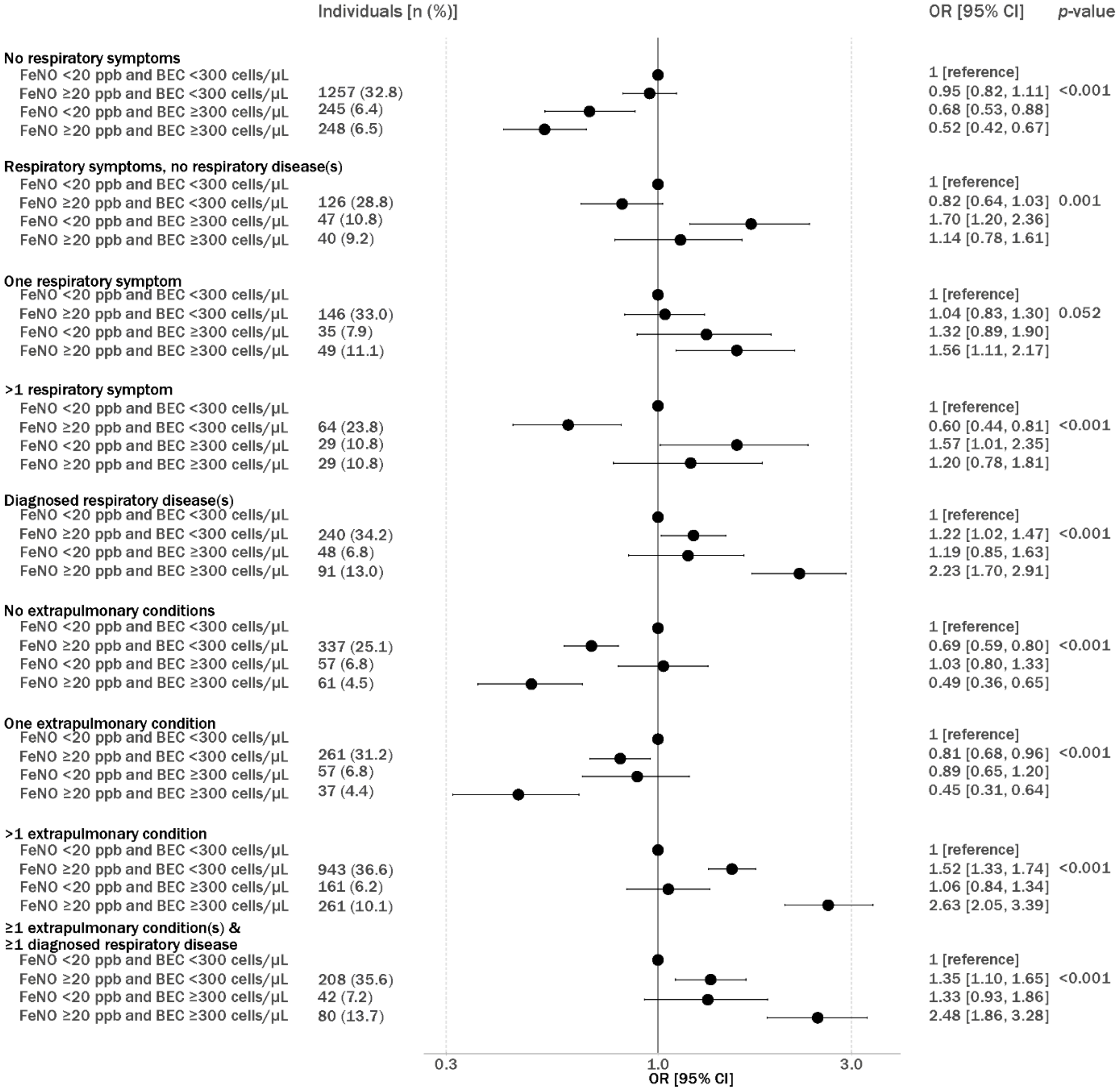

Figure S5 Odd ratios of elevated fractional exhaled nitric oxide (FeNO) levels and/or blood eosinophil counts (BEC) presenting respiratory and extrapulmonary conditions. Logistic regression models were used with normal biomarker levels (FeNO <20 ppb and BEC <300 cells/µL) as the reference group for all association and are adjusted for age and sex. Estimates are odds ratios (OR [95% confidence intervals]) where significance was considered where $p < 0.05$.
